# The human herpesvirus-type 8 is not involved in malignant melanoma

**DOI:** 10.1038/sj.bjc.6690322

**Published:** 1999-04

**Authors:** M Deichmann, M Thome, M Bock, A Jäckel, V Waldmann, H Näher

**Affiliations:** Universitäts-Hautklinik Heidelberg, Voβstraβe 2, 69115 Heidelberg, Germany

**Keywords:** malignant melanoma, human herpesvirus-type 8, interleukin-6, polymerase chain reaction

## Abstract

Malignant melanomas were supposed to harbour the human herpesvirus-type 8 (HHV-8) genome, as melanoma cells were reported to express interleukin-6 and a homologue of interleukin-6 was found in the HHV-8 genome. We therefore investigated 33 primary malignant melanomas by polymerase chain reaction, but could not find this tumorigenic gamma-herpesvirus in any tumour. © 1999 Cancer Research Campaign


*British Journal of Cancer* (1999) **80**(1/2), 67–69
© 1999 Cancer Research Campaign
Article no. bjoc.1998.0322

Short communication

### The human herpesvirus-type 8 is not involved in malignant melanoma

**M Deichmann, M Thome, M Bock, A Jäckel, V Waldmann and H Näher**

Universitäts-Hautklinik Heidelberg, Voβstraβe 2, 69115 Heidelberg, Germany

**Summary** Malignant melanomas were supposed to harbour the human herpesvirus-type 8 (HHV-8) genome, as melanoma cells were reported to express interleukin-6 and a homologue of interleukin-6 was found in the HHV-8 genome. We therefore investigated 33 primary malignant melanomas by polymerase chain reaction, but could not find this tumorigenic gamma-herpesvirus in any tumour.

**Keywords**: malignant melanoma; human herpesvirus-type 8; interleukin-6; polymerase chain reaction

The cytokine interleukin-6 (IL-6) is known as a growth factor in malignant melanoma (MM), stimulating tumour cells to proliferate (Kishimoto and Akira, 1992; Lee et al, 1992; Candi et al, 1997). Recently, a homologe to the human IL-6 has been identified in the human herpesvirus-type 8 (HHV-8) genome (Neipel et al, 1997). HHV-8 is a gamma-herpesvirus, related to the tumourigenic Epstein–Barr virus and herpesvirus saimiri and was first identified in AIDS-associated Kaposi's sarcoma (KS) in 1994 (Chang et al, 1994). HHV-8 has been detected consistently in HIV-related and HIV-unrelated cases of KS, but also in body cavity B-cell lymphomas and multicentric Castleman's disease (Cesarman et al, 1997; Nicholas et al, 1997). HHV-8-encoded IL-6 homologue (vIL-6) is the first known example of an IL-6-type gene in a viral genome (Neipel et al, 1997). The receptor binding region is highly conserved, and vIL-6 shares biological activities with human IL-6 protein (Nicholas et al, 1997).

The presence of a IL-6 homologue encoded by HHV-8 actually provides a mechanistic model for the hypothesized role of HHV-8 in KS, body cavity B-cell lymphomas and multicentric Castleman's disease, that involves the mitogenic effects of vIL-6 on surrounding cells. Transmitting this model to other tumours known to be stimulated by IL-6, the hypothesis was formulated that MM might also harbour HHV-8.

We therefore sought to identify HHV-8 DNA in melanoma tissue. Using a specific and highly sensitive polymerase chain reaction (PCR) protocol to amplify DNA of the viral minor capsid protein gene, we evaluated genomic DNA samples from 33 primary MMs for the presence of HHV-8 DNA.

### MATERIAL AND METHODS

### Isolation of genomic DNA from paraffin-embedded tissue

Genomic DNA was isolated from paraffin-embedded tissue from 45 MMs. All tumours were diagnosed with at least a thickness according to Breslow of 1.5 mm and Clark's level IV. Isolation of DNA was performed using the High Pure PCR template preparation kit (Boehringer Mannheim, Mannheim, Germany) according to the manufacturer's instructions. Quality of isolated DNA was assessed by 1% agarose gel electrophoresis.

*Received 26 June 1998*
*Revised 3 November 1998*
*Accepted 11 November 1998*

*Correspondence to:* M Deichmann

### Polymerase chain reaction

PCR was performed as described (Corbellino et al, 1996). HHV-8 DNA was amplified using primer pair 087se/088as (Corbellino et al, 1996), and 1 μl of the PCR products was amplified in nested PCRs, using primer pair KS330se/KS330as (Chang et al, 1994, Figure 1). DNA specimens from three Kaposi's sarcoma (KS) served as positive controls, water as negative controls. The absence of major *Taq* polymerase inhibitors, as well as DNA integrity in the tissue samples, were assessed by PCR amplification of a 268 bp human β-globin gene fragment, using the primers GH20 and PC04 (Greer et al, 1991). PCR products were analysed by 1.8% agarose gel electrophoresis.

### Sequencing of PCR products

KS330se/KS330as PCR products of 233 base pair (bp) length from KS tissue were separated electrophoretically on 1.8% agarose gel and purified with Qiaex II gel extraction columns (Qiagen, Chatsworth, CA, USA). DNA fragments (50–100 ng) were sequenced with Cycle Sequencing 9700 PE (Applied Biosystems, Foster City, CA, USA) using primers KS330se and KS330as respectively (Smith et al, 1986; Medigene, Martinsried, Germany). The samples were analysed with the ABI Prism 377 Genetic Analyzer (Perkin-Elmer). Sequences of PCR products were compared with HHV-8 DNA (EMBL data bank accession number KSV18551) using the softwares Clustal and BESTfit of the Heidelberg Unix Sequence Analysis Resources (HUSAR, version 4.0, DKFZ, Heidelberg).

### Sensitivity of the PCR assay

A 983 bp DNA fragment from KS DNA samples was amplified using primer pairs KS636se/KS1743as in a first and

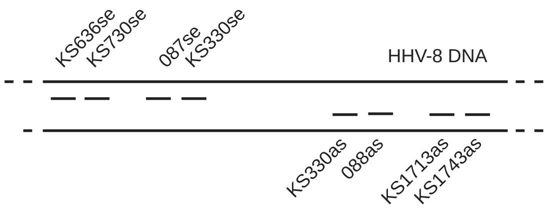

**Figure 1** Position of HHV-8-specific primers. The primer pair 087se/088as was used in a first and KS330se/KS330as in a nested PCR to investigate malignant melanoma DNA samples for the presence of HHV-8 DNA. The primer pair 636se/1743as was used in first and 730se/1713as in a nested PCR to synthesize high amounts of a 087se/088as spanning DNA fragment from the HHV-8 genome in Kaposi's sarcoma. Serially dilutions of this DNA fragment were assessed by the PCR assay used for melanoma investigation to estimate the sensitivity of this assay

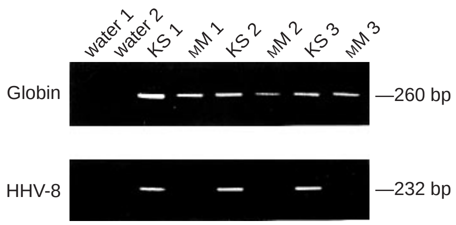

**Figure 2** HHV-8 DNA is not detected in malignant melanomas. Globin and HHV-8 PCR are shown one below another. Water served as negative controls, three Kaposi's sarcoma DNA specimens as positive controls (KS). In addition to the PCR results from three melanomas in this experiment (MM), 30 further tumours were tested negative

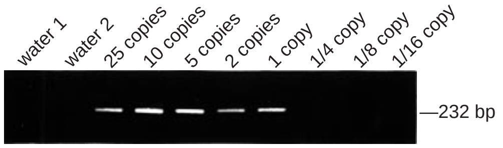

**Figure 3** Even a single copy of HHV-8 DNA was detected by the PCR protocol used for melanoma investigation. A DNA fragment spanning the 087se and 088as primer binding sites was synthesized, measured in quantity and serially diluted. Solutions containing 25, 10, 5, 2 and a single copy of this DNA fragment gave positive results in the HHV-8 PCR assay, whereas solutions expecting one-quarter, one-eighth and one-sixteenth copy on average were negative. Water served as negative controls

KS730se/KS1713as in a nested PCR (Figure 1). The sequences of the primers were: KS636se 5′-GCA CTC GAC AAG AGT ATA GTG G-3′; KS1743se 5′-ATA TCC ACG ATC CCG TCG CTG A-3′; KS730as 5′-TCG GAG ATT GCC ACC GTT TAC A-3′; KS 1713 as 5′-TCT TTG ATG GCG TCG GTC TCT A-3′. Numbers in the primers' designations are derived from HHV-8 minor capsid protein nucleotide sequence (Chang et al, 1994). PCR solutions and cycle conditions were the same as described (Corbellino et al, 1996). The 087se/088as spanning DNA fragment was cleaned using High Pure filter tubes (Boehringer Mannheim) according to the manufacturer's recommendations, measured by photometer five independent times and serially diluted. Solutions containing 25, 10, 5, 2, 1, one-quarter, one-eight and one-sixteenth copies of the 983 bp DNA fragment were assessed by the PCR assay used for HHV-8 DNA detection in melanomas.

### RESULTS

Before assessing melanoma DNA samples for the presence of HHV-8 DNA, size and quantity of isolated DNA were estimated by agarose gel electrophoresis, and the presence of amplifiable DNA was assessed by PCR amplification of a 268 bp human β-globin gene fragment. Thirty-three of 45 melanoma DNA samples (ten nodular melanomas, 15 superficial spreading melanomas, four acrolentiginous melanomas, two lentigo maligna melanomas, two subcutaneous metastases) were positive in globin PCR, and positive results in globin PCR were in accordance with agarose gel electrophoresis showing high molecular integrity of DNA.

Following globin PCR, melanoma DNA samples were assessed for the presence of HHV-8 DNA using primer pairs 087se/088as in a first, and KS330se/KS330as in a nested PCR to amplify a 232 base pair DNA fragment of the HHV-8 minor capsid protein gene. None of 33 melanoma DNA samples contained HHV-8 DNA (Figure 2). Assessing KS biopsies, all three DNA specimens were positive in HHV-8 PCR. Several water controls were all negative for HHV-8 DNA.

To confirm that HHV-8-DNA, but no related sequences, were amplified in the PCR reactions, cycle sequencing of PCR products was performed. The sequence of HHV-8 PCR products was determined for 230 nucleotides, which were identical with the HHV-8 DNA sequence from position 989 to 1219 (EMBL data bank accession no. KSV 18551).

To estimate the sensitivity of the PCR protocol used for melanoma testing, a DNA fragment spanning the 087se and 088as primer binding sites was synthesized from KS DNA samples. Diluting this 983 bp DNA fragment, even a single copy was detected by the HHV-8 PCR assay used for melanoma investigation (Figure 3). None of higher dilutions, e.g. one-quarter, one-eighth, and one-sixteenth copy of HHV-8 DNA, was positive in the HHV-8 PCR assay, making incorrect dilution unlikely.

### DISCUSSION

The recent identification of a vIL-6 gene in the HHV-8 genome suggested a putative role of this virus in the pathogenesis of MM: human melanoma cells have been reported to express IL-6 at mRNA and in part at protein level in vitro (Colombo et al, 1992; Lee et al, 1992; Francis et al, 1996). In addition, elevated IL-6 serum levels in patients with metastatic melanoma have been associated with resistance to immunotherapy and shorter median survival (Tartour et al, 1994, 1996) and are related to progressive disease (unpublished data). In accordance with these data, infection of melanoma cells by HHV-8 and transcription of vIL-6 was hypothesized to enhance MM tumour cell proliferation.

However, assessing genomic DNA samples from histologically proven MMs by a specific and highly sensitive PCR assay, HHV-8 DNA was not detected in any of 33 samples. The tumours were not investigated for IL-6 expression, which was assumed according to previous reports (Lee et al, 1992; Candi et al, 1997). We therefore conclude that melanoma tumour cell proliferation is unlikely to be enhanced by vIL-6 from HHV-8 infection. In the same way, elevated IL-6 serum levels related with progressive disease in metastasized patients are probably not caused by HHV-8 cytokine expression. Secretion of this protein more likely reflects acute-phase reaction caused by tumour disease, since IL-6 is known as a major molecular mediator of inflammatory conditions. IL-6 is

*© Cancer Research Campaign 1999*

produced by a variety of cells including fibroblasts, endothelial cells, keratinocytes, T and B lymphocytes and macrophages (Kishimoto, 1989; Klein et al, 1989).

Similar to malignant melanoma, IL-6 has been reported as an autocrine growth factor in KS and in multiple myeloma (Akira and Kishimoto, 1992; Suzuki et al, 1992), and elevated serum-IL-6 levels in multiple myeloma were associated with high tumour burden and with poor prognosis (DuVillard et al, 1995; Pelliniemi et al, 1995). Indeed, HHV-8 has been found in both diseases: HHV-8 was first detected in KS tissue (Chang et al, 1994) and has recently been identified in bone marrow from patients with multiple myeloma (Rettig et al, 1997; Said et al, 1997). HHV-8 encoded vIL-6 was transcribed in the myeloma bone marrow dendritic cells and therefore may be required for the growth of malignant plasma cells (Rettig et al, 1997). However, the role of HHV-8 in the pathogenesis of multiple myeloma remains controversial, as HHV-8 DNA was not found in mobilized blood mononuclear cells of patients with multiple myeloma (Cull et al, 1998).

In conclusion, IL-6 acts as an autocrine growth factor in MM similar to KS and multiple myeloma but is not of HHV-8 origin. Since this herpesvirus was recently detected, and is the first known example of a virus encoding a structural equivalent of IL-6 (Neipel et al, 1997), further viruses related to the tumourigenic gamma-herpesviruses known today will probably be identified and should be investigated for involvement in malignant melanoma and other tumours.

### ACKNOWLEDGEMENTS

The authors thank W Hartschuh for histologic evaluation of the malignant melanomas.

### REFERENCES

Akira S and Kishimoto T (1992) The evidence for interleukin-6 as an autocrine growth factor in malignancy. *Semin Cancer Biol* **3**: 17–26

Candi E, Knight RA, Spinedi A, Guerrieri P and Melino G (1997) A possible growth factor role of IL-6 in neuroectodermal tumours. *J Neurooncol* **31**: 115–122

Cesarman E and Knowles DM (1997) Kaposi's sarcoma-associated herpesvirus, a lymphotropic human herpesvirus associated with Kaposi's sarcoma, primary effusion lymphoma, and multicentric Castleman's disease. *Semin Diagn Pathol* **14**: 54–66

Chang Y, Cesarman E, Pessin MS, Lee F, Culpepper J, Knowles DM and Moore PS (1994) Identification of herpesvirus-like DNA sequences in AIDS-associated Kaposi's sarcoma. *Science* **266**: 1865–1869

Colombo MP, Maccalli C, Mattei S, Melani C, Radrizzani M and Parmiani G (1992) Expression of cytokine genes, including IL-6, in human malignant melanoma cell lines. *Melanoma Res* **2**: 181–189

Corbellino M, Poirel L, Bestetti G, Pizutto M, Aubin JT, Capra M, Bifulco C, Berti E, Agut H, Rizzardini G, Galli M and Parravicini C (1996) Restricted tissue distribution of extralesional Kaposi's sarcoma herpesvirus-like DNA sequences in AIDS patients with Kaposi's sarcoma. *AIDS Res Hum Retroviruses* **12**: 651–657

Cull GM, Timms JM, Haynes AP, Russel NH, Irving WL, Ball JK and Thomson BJ (1998) Dendritic cells cultured from mononuclear cells and CD34 cells in myeloma do not harbour human herpesvirus 8. *Br J Haematol* **100**: 793–196

DuVillard L, Guiguet M, Casasnovas RO, Caillot D, Monnier-Zeller V, Bernard A, Guy H and Solary E (1995) Diagnostic value of serum IL-6 level in monoclonal gammopathies. *Br J Hematol* **89**: 243–249

Francis GM, Krohn EG, Woods KV, Buzaid AC and Grimm EA (1996) Interleukin-6 production and secretion in human melanoma cell lines, regulation by interleukin-1. *Melanoma Res* **6**: 191–201

Greer CE, Peterson SL, Kiviat NB and Manos MM (1991) PCR amplification from paraffin-embedded tissues, effects of fixative and fixation time. *Am J Clin Pathol* **95**: 117–124

Kishimoto T (1989) The biology of interleukin-6. *Blood* **74**: 1–10

Kishimoto T and Akira S (1992) Interleukin-6 and its receptor, a paradigm for cytokines. *Science* **258**: 593–597

Klein B, Zhang XG, Jourdan B, Content B, Content J, Houssiau F, Aarden L, Piechaczyk M and Bataille R (1989) Paracrine rather than autocrine regulation of myeloma-cell growth and differentiation by interleukin-6. *Blood* **73**: 517–526

Lee JD, Sievers TM, Skotzko M, Chandler CF, Morton DL, McBride WH and Economou JS (1992) Interleukin-6 production by human melanoma cell lines. *Lymphokine-Cytokine-Res* **11**: 161–166

Neipel F, Albrecht JC, Ensser A, Huang YQ, Li JJ, Friedman-Kien AE and Fleckenstein B (1997) Human herpesvirus 8 encodes a homolog of interleukin-6. *J Virol* **71**: 839–842

Nicholas J, Ruvolo VR, Burns WH, Sandford G, Wan X, Ciufo D, Hendrickson SB, Guo HG, Hayward GS and Reitz MS (1997) Kaposi's sarcoma-associated human herpesvirus-8 encodes homologues of macrophage inflammatory protein-1 and interleukin-6. *Nat Med* **3**: 287–292

Pelliniemi TT, Irjala K, Mattila K, Pulkki K, Fajamaki A, Tienhaara A, Laakso M and Lahtinen R (1995) Immunoreactive interleukin-6 and acute phase proteins as prognostic factors in multiple myeloma, Finnish leukemia group. *Blood* **85**: 765–771

Rettig MB, Ma HJ, Viscio RA, Pold M, Schiller G, Belson D, Savage A, Nishikubo C, Wu C, Fraser J, Said JW and Berenson JR (1997) Kaposi's sarcoma-associated herpesvirus infection of bone marrow dendritic cells from multiple myeloma patients. *Science* **276**: 1851–1854

Said JW, Rettig MR, Heppner K, Vescio RA, Schiller G, Ma HJ, Belson D, Savage A, Shintaku IP, Koeffler HP, Asou H, Pinkus G, Pinkus J, Schrage M, Green E and Berenson JR (1997) Localization of Kaposi's sarcoma associated herpesvirus in bone marrow biopsy samples from patients with multiple myeloma. *Blood* **90**: 4278–4282

Smith LM, Sanders JZ, Kaiser RJ, Hughes P, Dodd C, Connell CR, Heiner C, Kent SBH and Hood LE (1986) Fluorescence detection in automated DNA sequencing analysis. *Nature* **321**: 674–679

Suzuki H, Yasukawa K, Saito T, Goitsuka R, Hasegawa A, Ohsugi Y, Taga T and Kishimoto T (1992) Anti-human interleukin-6 receptor antibody inhibits human myeloma growth in vivo. *Eur J Immunol* **22**: 1989–1993

Tartour E, Dorval T, Mosseri V, Deneux L, Mathiot C, Brailly H, Montero F, Joyeux I, Pouillart P and Fridman WH (1994) Serum interleukin 6 and C-reactive protein levels correlate with resistance to IL-2 therapy and poor survival in melanoma patients. *Br J Cancer* **69**: 911–913

Tartour E, Blay JY, Dorval T, Escudier B, Mosseri V, Douillard JY, Deneux L, Gorin I, Negrier S, Mathiot C, Pouillart P and Fridman WH (1996) Predictors of clinical response to interleukin-2-based immunotherapy in melanoma patients, a French multiinstitutional study. *J Clin Oncol* **14**: 1697–1703

© Cancer Research Campaign 1999 

